# NASA resilience and leadership: examining the phenomenon of awe

**DOI:** 10.3389/fpsyg.2023.1158437

**Published:** 2023-06-09

**Authors:** Jeff Thompson

**Affiliations:** ^1^Columbia University Irving Medical Center, Columbia University, New York, NY, United States; ^2^Lipscomb University, Nashville, TN, United States

**Keywords:** awe, leadership, resilience, NASA, phenomenology, meaning in life, well-being

## Abstract

This study examines how a phenomenon, awe, along with related resilience practices, is perceived by a NASA medical and mental health professional, who also serves in a leadership role, and how awe has impacted their work and personal life. Considering both their leadership role and how their work involves supporting the wellbeing of astronauts pre-mission, during missions, and post-mission, the potential impact of awe on the NASA expert has individual implications along with many others, especially in stressful environments. The results indicate that reflecting on awe experiences can support a person finding meaning and purpose in their life, evoke gratitude, increase social connectedness, promote optimism and other resilience skills in the moment, and generally have a sustainable positive effect.

## Introduction

Medical professionals and healthcare workers must maintain a sufficient level of personal wellbeing and resilience in order to effectively support the wellbeing of others. A key element of sustaining and enhancing personal resilience is finding meaning and purpose in both life and work while also engaging in other evidence-based resilience practices that can be utilized in a practical manner by these professionals. When viewed as a resilience practice, engaging in a practice of reflecting on and sharing awe experiences can support the wellbeing of medical professionals and healthcare workers.

This phenomenological study demonstrates that eliciting awe experiences from a National Aeronautics and Space Administration (NASA) leader, who is also a mental health and medical expert responsible for the wellbeing of astronauts, can support a person’s resilience by providing meaning and purpose in life, evoking gratitude, enhancing social connectedness, and promoting optimism and other related resilience attributes. Given the high-pressure, time-consuming nature of their work, professionals such as the NASA expert who took part in this study require minimally time-consuming, evidence-based practices.

Generally speaking, recent research alarmingly demonstrates the necessity of resilience and wellbeing practices as the majority (90%) of Americans feel the country is facing a mental health crisis (Panchal et al., 2023). According to a review by the National Institute for Occupational Safety and Health (NIOSH), healthcare workers have expressed feeling physically and emotionally exhausted, stressed, and unable to obtain enough support; they feel as if they are stretched too thin and many are experiencing burnout (Mental Health America, n.d.). Further, data shows that 69% of physicians experienced depression, while 13% had suicidal thoughts (NIOSH, 2022). Psychiatrists have reported burnout rates at 78% (Summers et al., 2020), while a study by Dawar and colleagues (2021) obtained worrying results showing that medical professionals struggle with maintaining a work-life balance, experience an overall high burnout, have sleep issues, and suffer from emotional exhaustion. These negative repercussions are not limited to medical professionals and can directly impact patients as a result of compassion fatigue, among other concerns (Clay, 2022), and can also adversely affect their personal lives (Olazagasti et al., 2021).

Addressing these issues is the responsibility of both the medical and mental health professional, while their organization is also responsible for doing so. Organizations can provide proactive, resilience, and wellbeing programs, although as these are frequently voluntary, the individual must also proactively take part in them.

The following sections provide an overview of the existing awe literature, illustrating how awe can support a person’s wellbeing as well as other resilience practices such as enhancing a person’s sense of meaning and purpose in life.

## Awe

Awe has been described as a complex emotion that captivates one’s attention when in the presence of something or someone extraordinary and challenges one’s current thinking (Stellar, 2021; Thompson, 2022a,b). Awe is often felt along with other positive emotions and therefore can support a person’s resilience and overall wellbeing (Thompson, 2022c; Thompson et al., 2022). Awe is a subjective experience (Graziosi, 2018); however, awe can be evoked by nature and space, music and the arts, religion and spirituality, social connectedness, and both personal accomplishments and those of others (Shiota et al., 2007; Allen, 2018; Graziosi and Yaden, 2019; Yaden et al., 2019; Sturm et al., 2020; Thompson, 2022c).

Awe is generally experienced as a positive emotion. In this context, awe can be supportive of a person’s wellbeing in a variety of ways. It increases their altruism, curiosity, creativity, critical thinking, empathy, focus, generosity, and gratitude, helps them handle ambiguity and uncertainty, makes them feel humble (“small” in a good way), and enhances their life satisfaction, problem-solving skills, and social connectedness (for a review, see Allen, 2018 and Thompson et al., 2022). Experiencing awe can also reduce impatience, stress, and make personal problems seem less overwhelming. Awe has also been described as a self-transcendent emotion, as the experience can lead to feeling more connected to others and can increase prosocial behaviors (Piff et al., 2015; Bai et al., 2017; Thompson, 2022a).

Meng and Wang (2022) have shown that prosocial behaviors increased when awe was elicited in the context of the workplace. Additionally, Thompson and Jensen’s (2023) work on awe and the workplace showed how reflecting and sharing work-related awe experiences can support police hostage negotiators’ wellbeing, and also demonstrated the direct relationship between awe and negotiator effectiveness. Those findings are relevant to this study in two ways. First, they demonstrate how reflecting on awe narratives can support a person in their work and in their personal wellbeing. Second, this study, which is part of a larger one with additional NASA personnel, utilized a design similar to the one conducted with the hostage negotiators.

Awe can also be elicited in a variety of ways aside from direct, in-person experience. Awe can also be experienced through augmented and virtual reality, pictures, video and audio clips, and narratives (Rudd et al., 2012; Piff et al., 2015; Chirico et al., 2016, 2017; Bai et al., 2017; Danvers and Shiota, 2017; Krenzer et al., 2018; Stellar et al., 2018; Chen and Mongrain, 2020; Cuzzolino, 2021; Walker and Gilovich, 2021; Thompson, 2022c). Awe narratives are the focus of this study, as sharing narratives can support finding meaning and purpose in life and can also elicit awe and other positive emotions in the reader, viewer, or listener (Shiota et al., 2007; Piff et al., 2015; Bai et al., 2017; Danvers and Shiota, 2017; Stellar et al., 2018; Chen and Mongrain, 2020; Cuzzolino, 2021; Thompson, 2022a,c,d; Thompson et al., 2022). The impact of reflecting on and sharing narratives is further explored in the narrative medicine section.

Experiencing awe has been described as both a mindfulness and resilience practice (Sturm et al., 2020; Tabibnia, 2020; Thompson, 2022d). With terms of mindfulness, awe can be viewed as being more accessible as it can be experienced automatically, while other mindfulness practices require training (D’Ardenne, 2019). As a resilience practice, experiencing and reflecting on awe are closely associated with finding meaning and purpose in life (Li et al., 2019; Rivera et al., 2019; Thompson, 2022c) as well as with other resilience practices (Tabibnia, 2020; Thompson et al., 2022).

**Figure 1:**
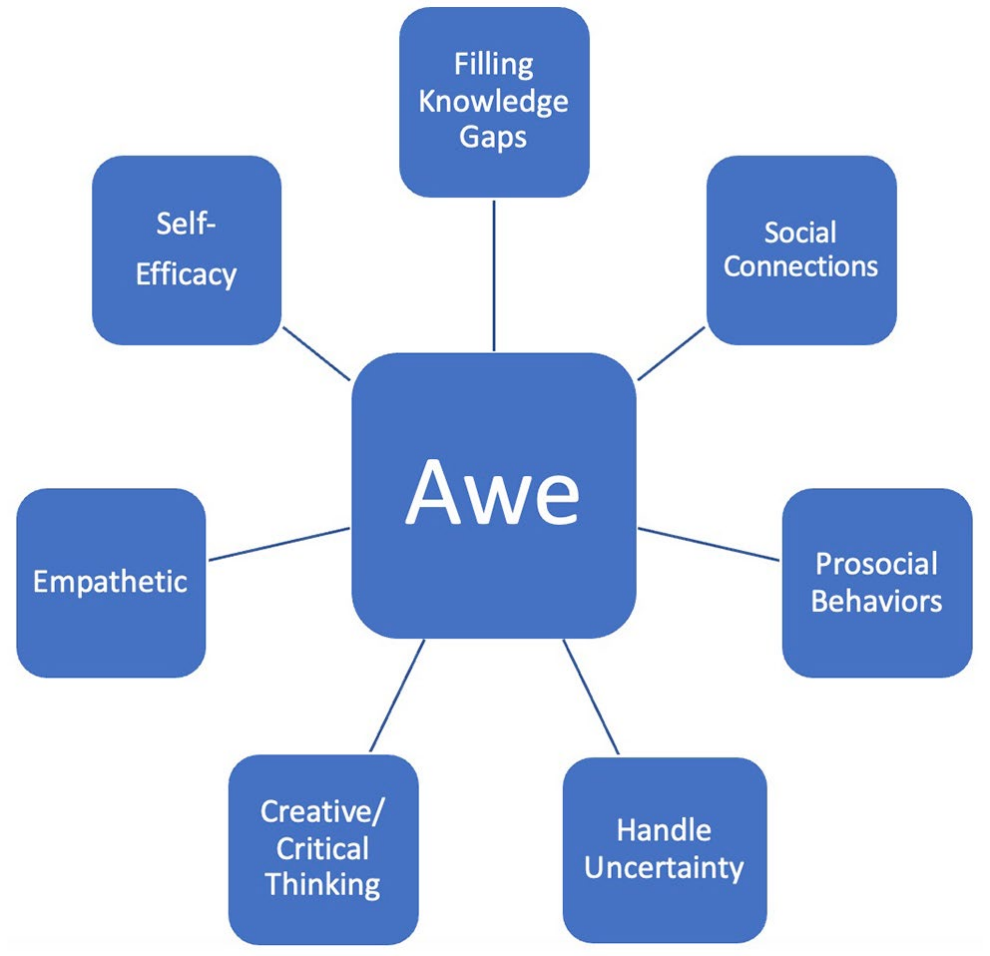
Awe A “Gateway” to supporting resiliences and leadership.

Figure 1 demonstrates how reflecting on and experiencing awe can serve as a gateway to supporting other resilience practices that also correspond with established leadership traits.

Similar work has theorized this awe-leadership connection (Thompson, 2022b) while exploratory studies have also begun to examine this “gateway” role awe can serve in an individual’s professional and personal life (Thompson, 2023a). The relationship between awe and resilience is further discussed in the next section.

## Resilience

The definition of resilience continues to evolve. Based on recent resilience literature (Thompson and Jensen, 2023), the author defines resilience as a set of practices that are proactive, on-going, and responsive to stressful and adverse events that sustain and enhance one’s mental health and overall wellbeing. Importantly, practicing resilience involves not only preparing, enduring, and recovering from negative events, but also includes purposefully planning, experiencing, and reflecting on positive moments (Tabibnia, 2020; Thompson, 2020; Thompson and Drew, 2020).

George Bonanno’s work on resilience (2005) emphasizes the importance of having a variety of practices to rely on and utilize based on a situation’s context. Thompson et al. (2022) provide a review of common resilience practices and their work has guided the development of this study. The resilience practices related to this study include cognitive reappraisal, connectedness, curiosity, gratitude, humility, meaning and purpose in life, and both prospection and optimism.

One resilience practice, for example finding meaning and purpose in life, can often create an “upward spiral,” as it can incorporate other resilience practices and positive emotions to contribute to living a mentally and physically healthy life (Korb, 2015).

Having meaning and purpose in life (MPiL) refers to feeling as if one’s life has an overall purpose and significance. Additionally, helping others, pursuing personal goals, and having order in life can contribute to having meaning and purpose (Baumeister et al., 2013; Tongeren et al., 2016; Martela et al., 2018; Routledge and FioRito, 2021; Huang and Yang, 2022). The upward spiral benefits of MPiL are numerous and can include improved sleep, social connectedness, prolonged life, and managing daily stressors (Routledge & FioRito; Williams, 2019; Thompson et al., 2022).

Based on its attributes, MPiL and its relationship with other resilience practices can support countering “downward spiral” common negative thoughts frequently associated with suicide, such as thwarted belongingness, hopelessness, and helplessness (Joiner, 2005; Van Orden et al., 2010, 2012; Thompson, 2022e). For instance, employees that feel a sense of belonging at work experience a greater feeling of overall wellbeing (Herbert, 2022). The same data showed that the benefits to an organization when an employee experiences that sense of belonging include greater work performance, higher productivity, greater retention, and increased revenue. A key contributor to providing that sense of meaning and belonging in the workplace was the organization’s leadership and the employee’s manager.

Kim and colleagues’ (2022) recent research showed how watching a short nature-based video can evoke awe and enhance finding meaning in life. Thompson’s work has also shown how watching short, awe-related videos can have the same impact, one participant sharing the following: “I knew the earth was so small but did not realize how small, wow! Even small, we have so much beauty and so much to offer” (2022c, p. 103).

## Leadership and reflection practices

Reflective practices have been shown to support people’s overall wellbeing, especially in challenging and complex conditions (Strumm, 2022). This includes contributing to enhancing many of the previously mentioned resilience attributes that are also indicative of effective leadership (Thompson, 2022a,b; Thompson and Jensen, 2023). In the health care profession, reflective practices have been suggested to support leadership and overall skills, wellbeing, and mental health (Frugé and Drutz, 2009; Koshy et al., 2017; Agnew, 2022).

Finally, and with direct relevance to this study, self-reflection has been described as a practice that can enhance a leader’s competency (Goleman et al., 2002). Additionally, guided reflective practices have been suggested as a method of developing leadership skills (Roberts, 2008) while they can also be supportive of psychological wellbeing, social connectedness, purpose in life, and self-efficacy (Czyżowska and Gurba, 2021).

## Methodological framework

Qualitative methodologies, specifically phenomenology, interpretative phenomenological analysis, and narrative medicine, directed the development of this study. This study explores how awe, when viewed as a resilience practice, can support the skills and overall resilience of a NASA leader and medical expert. Therefore, the guiding question of this study was, “How does a specific phenomenon, awe, support a NASA leader and mental health professional with the work they do and in terms of their wellbeing?” Because a phenomenon was being examined, phenomenology was primary qualitative methodology utilized, including specifically interpretative phenomenological analysis.

### Phenomenology and interpretative phenomenological analysis

Phenomenology was the leading methodology that guided the study, as its purpose is to understand how a person experiences a phenomenon, in this case awe (van Manen, 1990; Neubauer et al., 2019; Thompson, 2022d). Phenomenology is examined in three parts: the specific experience of a phenomenon, how a person (or group) experiences that phenomenon, and the specific context in which it is experienced (Thompson, 2022d). This study examined how a specific person, a NASA medical expert, research scientist, and leader, experiences awe in relation to their work and personal life. As phenomenology is concerned with quality over quantity, a large sample is not indicative of a rigorous study. Rather, “thick descriptions” are utilized, whereby meaning is derived and themes are established based on detailed accounts of the participant (Geertz, 1973; Lincoln and Guba, 1985; Holloway, 1997; Mills et al., 2010). The sample size selection is further explained below.

Interpretative Phenomenological Analysis (IPA) also supported this study in a variety of ways, as IPA examines how a person makes sense of their world, especially when it is complex and involves novelty (Smith and Osborn, 2004). IPA is a research methodological approach that explores (1) how a person makes meaning of a phenomenon they have experienced and (2) how the researcher then interprets that experience and the meaning shared by the person. Importantly, this demonstrates the pivotal role of the researcher. IPA does not solely involve gathering and sharing a narrative; rather, the research is responsible for interpreting the various statements (or parts) while also taking into consideration what else has been shared (the whole), thus allowing for patterns and meaning to emerge (Guba and Lincoln, 1994; Smith and Osborn, 2003; Smith et al., 2009). This process has been called the hermeneutic circle (Frechette et al., 2020), whereby, based on the researcher’s experience of the phenomenon along with the analysis of the data, themes can be established (Smith et al., 2009).

With respect to the sample size, IPA researchers have broadly explained that the design of a study is not prescriptive; instead, each researcher is supported by guidelines and suggestions adapted and modified for the purpose of their study (Smith and Osborn, 2004). For this particular study, which is part of a larger research study of NASA employees with responsibilities similar to those of this participant, a single participant’s understanding of awe is being examined. Although qualitative studies utilizing phenomenological methods often comprise multiple participants, the goal once again is quality over quantity. The aim is to understand how an individual understands the phenomenon. Smith and Nizza (2021) explain how “a single case study can yield rich and interesting results” (p. 15), while according to Smith and colleagues (2009), a single case can be “especially powerful” (p. 51). Smith and Osborn (2004) remind researchers (and readers) that there is no right answer to sample size while one is acceptable, especially as IPA is an “in-depth qualitative analysis” (p. 54).

### Narrative medicine

Rita Charon, MD, a proponent of narrative medicine, explains it as model for humane and effective medical practice (Charon, 2001), which includes a commitment to understanding patients’ lives, caring for the caregivers, and giving voice to the suffering (Krisberg, 2017). This study embraces the caregiver’s side of narrative medicine. Further, a fundamental aspect of narrative medicine is understanding meaning through the stories that we tell (Zaharias, 2018), as it is argued that this can improve patient care (Krisberg, 2017). More generally, story-telling is also an established practice in positive psychology because it can help listeners understand complex topics while also providing meaning for them in personal ways (Suzuki et al., 2018). It is important to point out that story-telling is not only supportive of the wellbeing of the listener, but also that of the sharer. Elaborating on this point, Charon (2001) explains that in order to support others in a medical and mental health role, the professional must look inward. Thompson recently explored this idea specifically in relation to awe narratives and mindfulness, suggesting that both sharing an awe story and being the recipient of that story can support a person’s resilience and overall wellbeing (Thompson, 2022d, 2023b).

Launer’s (2002) seven underpinnings to narrative medicine further establish how the narrative medicine approach complements other methodologies and the specific design and intention of this study. For example, one of the seven underpinnings is curiosity. Awe research has been shown to promote curiosity, and curiosity supports other resilience practices such as finding meaning and purpose in life (Thompson, 2022c; Thompson et al., 2022). Launer believes that curiosity is necessary for medical professionals to effectively support their patients.

This study argues that the concepts of narrative medicine should be expanded beyond medical professionals and be embraced by all professionals or practitioners engaged in providing mental health support. Considering this study’s participant works in the medicine and mental health field at NASA while also in a leadership role, the concepts and goals of narrative medicine align with the scope of this study and the expression “narrative medicine” can be viewed as more broadly as narrative (mental) health.

## Methodology

### Design

The previous section details the various research methodologies that guided the development of this study to examine a specific element of resilience, the phenomenon of awe. This section explains the specific design of the study.

#### Semi-structured interview

This form of interviewing, consistent with phenomenological research, allows the researcher and participant to engage in a dialogue whereby initial questions are modified in the light of the participants’ responses and the investigator is able to probe interesting and important areas that arise (Smith and Osborn, 2003; Thompson, 2015; Smith and Nizza, 2021; Thompson and Jensen, 2023).

The semi-structured interview, which was conducted and recorded *via* Zoom, comprised approximately 19 questions and lasted approximately 1 hour. Consistent with qualitative research, especially phenomenology, the questions were designed to guide the conversation and were not all necessarily asked in a specific order (Smith and Nizza, 2021). Some questions were also not asked either because they were answered without being prompted, or deemed not required by the interviewer (the author) because they were unnecessary or due to time constraints.

Lastly, consistent with previous research suggestions and practices (Cuzzolino, 2021; Thompson, 2022c), a follow-up survey was sent to the participant 5 days later to gather insights with respect to taking part in the study.

#### The participant

The participant serves in a leadership role and is responsible primarily for the mental health and overall wellbeing of current astronauts as well as their families. The participant, who has worked at NASA for many years, is well-respected and an expert in this field of research and practice. To provide further information on the participant would put their anonymity at risk, which is why the participant’s gender is being de-identified and plural pronouns (they and them) used instead. The participant was specifically selected to take part in the study based on the participant’s role in NASA. They were contacted *via* email and, prior to the interview, provided consent to participate. The study was approved through the author’s institutional review board at Lipscomb University.

#### Data analysis

The interview was transcribed and previous research, specific to awe and resilience, guided data analysis, development of themes, and presentation of themes (Bonner and Friedman, 2011; Cuzzolino, 2021; Thompson, 2022c,d; Thompson and Jensen, 2023). Consistent with phenomenology and specifically IPA, the author’s understanding and previous work on resilience and awe, and in analyzing the data from this study, themes emerged and are outlined in the next section.

## Results

The analysis of the data collected from the interview resulted in the emergence of multiple themes, many of them consistent with previous awe research as more broadly about resilience (see Thompson, 2022c; Thompson et al., 2022; Thompson and Jensen, 2023). As described above, the themes emerged through data analysis in a manner consistent with various qualitative methodologies, mainly IPA. Previous awe research (Bonner and Friedman, 2011; Cuzzolino, 2021; Thompson, 2022c; Thompson and Jensen, 2023) provided a further framework in which to analyze the data for the purpose of identifying themes.

### Themes

The themes and examples listed in Table 1 expand on the relationship between awe, resilience, and leadership that was shared in Figure 1. In Table 1, each theme, included those shared in Figure 1, is supported by participant quotes in a similar manner to previous qualitative studies on awe and resilience (e.g., Thompson and Drew, 2020; Thompson, 2022c) as well as being consistent more broadly with IPA (Smith et al., 2009; Smith and Nizza, 2021).

**Table 1:** Awe related themes.

Theme (listed alphabetically)	Example
Accomplishments (self-efficacy)	*What I have been a part of is still going to be looked at as being a pioneer in the space program. Multiple space missions that I’ve been personally a part of are the first that have ever been done.*
Cognitive reappraisal	*One day I left here and I was having a terrible day as we all do, right? … And when I got there [somewhere else where they were doing volunteer work] and listening to what they are going through and knowing that I was there to actually lend myself to them, I suddenly felt great again.*
Connectedness	*It makes you feel that you are part of something great.*
Curiosity	*I cannot wait to talk to them and to hear how has the last year gone for them.*
Gratitude	*Gratitude I think go hand in hand [with awe].*
Humility	*I realized, [Hey, I’m not the smartest person in the room almost ever anymore]. And I tell you it’s been a blessing; it’s just been incredible.*
Intensity	*They are very intense [awe experiences].*
Learning/filling knowledge gaps	*[Awe] inspires you to want to learn more.*
Meaning and purpose in life	*I’m doing something that’s way beyond just coming here to get paid, way beyond that.*
Mindfulness	*The freedom from being worried or being concerned about the future or the past.*
Motivating	*Inspires… to do more.*
Nature	*It could be a moment when you are hiking… with me, it’s primarily out when I’m outdoors.*
“Ordinary” moments	*There are times in which I’ll either drive into work or leave work and it might be just a regular day… But going into work and seeing the big sign, the [NAME] Space Center… I still love working here.*
Open-mindedness	*I have a broader view and understanding of awe.*
Physical sensations	*I could make myself feel tearful thinking about all of the opportunities I’ve had.*
(Other) positive emotions	*Makes you feel wonderful.*
Profound	*In a good sense of exhilaration.*
Prospection and optimism	*I could picture easily working here another decade, easy, because it’s not a job that punishes you… It actually makes you feel better about yourself.*
Rare moments	*I found out that this is a once in a lifetime opportunity. I could work anywhere else I want to and all those jobs are always going to be open*
Self-care	*Now I really pay more attention to making certain that I sleep adequately.*
Self-transcendent experience	*I’m building the foundation of something that’s going to be long lasting and not just going to just go away.*
Small	*The people that now I supervise are brilliant. Everyone is dedicated to the common cause of something greater than ourselves.*
Spiritual	*Thinking to myself [this has] that religious feeling.*
Uncertainty and ambiguity	*Awe – it fills you up, but fills you up in a way that does not strike your autonomic nervous system in a jarring way. It’s relaxing.*

Awe has previously been described as a complex emotion, as illustrated in the themes displayed in Table 1. First, the numerous (24) themes that emerged demonstrate the complex nature of the phenomenon. With respect to these themes, previous research has emphasized that phenomenology is not limited to a strict, set number of themes, but rather the examination of the phenomenon and subsequent themes are determined by the purpose of the study and the analysis of the researcher (Creswell, 2007; Smith and Nizza, 2021; Thompson, 2022c).

Next, the complexity of awe is demonstrated in each of the themes, as often they are not independent and isolated from the other themes. Rather, their complexity is revealed through their interconnectedness. For example, the quotation provided to support “prospection and optimism” also includes elements of gratitude, connectedness, and having meaning in purpose in life, specifically regarding their work.

Having presented the themes related to awe, we can now expand on them narratively, with support from the participant’s comments. The section concludes with discussing the practical implications this awe-based interview had on the participant.

### Awe interview response

The interview began with gathering basic demographic data and information related to the work they are involved in. Immediately, numerous connections to awe and resilience were revealed when the participant described what it was like when first beginning to work at NASA and continuing to work there many years later:

I took the chance, I took the pay cut, took the chance—a once-in-a-lifetime opportunity. It was difficult at first, like a fish out of water. I realized, hey, I’m not the smartest person in the room almost ever anymore. And I tell you it’s been a blessing; it’s just been incredible.

I could make myself feel tearful thinking about all of the opportunities I’ve had. I’ve traveled all over the world. I work directly with astronauts. I’ve been to multiple space flight launches and landings. The people that now I supervise are brilliant.

Everyone is dedicated to the common cause of something greater than ourselves. Everyone that I work with can make more money somewhere else. So, in short, yeah, it’s been a remarkable experience. I am extremely grateful.

When people tell you NASA is the best place to work, it’s not an exaggeration: it really is.

In this short, opening description of their work, the NASA expert shared elements related to both awe and resilience, including a sense of accomplishment, connectedness, gratitude, humility, meaning and purpose in life, profoundness, and handling uncertainty. They then added further information about their specific work:

We [primarily NASA psychiatrists and psychologists] work directly in support of human space flight missions. We do engage in some research … our group works directly with astronauts … providing them with the services that they need to optimally fly a human space flight mission.

Not just get by … but how to perform optimally under the most difficult circumstances.

These are high performing individuals [the astronauts]. [Our job is] to get people, not only [to] perform their best, but also when they come back from the mission to not be worn out, or burnt out.

The NASA expert further explained their group’s work related to the astronauts:

And so, our job is to select them, engage in astronaut selection, training, preflight services, preflight assessment, inflight services and assessment, and then post-flight. And then, use all that information to make it better and continually better, continuous quality improvement over decades with the goal eventually being the lunar missions and then a Mars mission.

I think that the key is [that] in order to work in our area, you have to feel comfortable working with high performing individuals.

Their statement importantly identifies that it is not only the astronauts that must be able to work in a high-pressure environment, but also the NASA health expert leader and their team as well. In order for their team to be effective, they explained how teamwork and other related attributes are required:

We’ve always had a high degree of collegiality, collaboration, and supportiveness. We support each other, defend each other, back each other up, watch out for each other, and educate each other.

I would say that it’s important for team members to know each other professionally and personally, and to have a high regard for one another—not just show up for work.

The NASA leader elaborated further on creating an environment that contributes to providing meaning and purpose to the work they do together:

At least before the pandemic, it was common for us to have lunch together, to socialize together, travel internationally together. And so, I think that’s very, very helpful. If you go to work and you just know the people, “Hey, this is Dr. so-and-so, that’s Dr. so-and-so,” but if it does not really go beyond that, I think that you are missing something in your life.

… I’ve had jobs where everything was professional and there might be some collegiality, but it really did not go beyond that. I think then when it does and you are an organization [in which] it does, it actually makes it more special. At our organization, we like to look at it as [if] we are a family.

I think it’s easier to perform with true superior quality if you are working with people that you feel passionately about supporting, and really enjoy each other’s company.

The discussion continued on how to develop an effective team environment. In reality, however, it’s not always possible. They explained how this was the case in their previous job:

The hardest place I ever worked … it was a very difficult place to work. And I saw people that were there to put their 30 years in and were completely miserable, completely miserable. And it was a miserable job, but at the same time they bought into that.

The NASA health professional’s previous statement identifies a critical component to resilience—perspective—and how having a certain perspective is necessary to effectively handle non-ideal environments in a healthy manner. Resilience researcher Dr. Rick Hanson describes this concept as having agency and “being the hammer, not the nail” (2018, p. 79). Further, it entails controlling what you can and coming to terms with what you cannot. Embracing this type of resilient mindset can lead to an upward-spiral of other resilience practices, including being optimistic.

The interview then transitioned to the phenomenon being examined: awe. When asked to define “awe,’ the NASA expert shared candidly:

Oh gosh. I should have looked it up. I’m glad I did not.

Well, I used the word awesome a lot, but awesome obviously comes from awe. And my layman’s version of awe would be something, anything, whether it’s something that you are looking at, something that you read, something that you feel, something that you are doing that makes you feel wonderful.

It makes you feel that you are part of something great that’s inspiring—that inspires you to want to learn more, to do more. It makes you feel more passionate about something or just makes you feel wonderful.

Their definition of awe is consistent with much of the literature on awe. As explained by our NASA expert, awe can be elicited in a variety of ways; it creates powerful and profound additional positive emotions, generates a sense of connectedness, provides meaning and purpose, and motivates a person to fill knowledge gaps. Moreover, awe provokes action.

As their narrative related to awe begins to unfold, it is important to state here how initially the participant displayed immediate (although it was pervasive throughout the interview) humility. This is notably important given the work they are engaged in, and especially in a leadership role while also being part of the team that is responsible for the wellbeing of astronauts. There are a distinct, limited group of people in the United States who are able to say their work is of national interest and importance. The NASA expert is part of this privileged group, where it is arguably much more than that: their work is of global significance. As will be discussed later, because of their important role and work at NASA, gaining insight into the impact of awe related to their work and in their personal life has significant value.

After sharing their definition of awe, they offered the following as a general example:

It could be a moment when you are hiking and you reach the peak of a mountain and you are looking around at all the other peaks.

The space, medical, and mental health expert followed this generalized example of awe with a personal experience that was vividly shared, even though it had occurred many years previously:

I still remember snorkeling … you can just wade in from the shore, crystal clear water; put on a snorkel and your fins and then you go out into this preserve and suddenly there’s this crater that goes from water of eight feet deep and suddenly goes down to 50 feet. [The water is] so clear. I remember I saw sea turtles, moray eels, and coral and thinking to myself [that this has] that religious feeling.

Awe has been described as a complex emotion and the above experience illustrates this. Being in nature evoked awe for them, but also present was a profound spiritual aspect as well. This deep and spiritual relationship could be what makes this memory so vivid for them, even though the event occurred so many years ago.

Furthermore, and adding to the spirituality of the moment, the NASA executive included additional common attributes associated with experiencing awe: a sense of smallness and of being part of something larger than themselves that creates a powerful sense of connectedness:

It struck me that this cannot all be just chance that this is here like this, the beauty of it, the interconnectedness of it, this whole ecosystem. And that feeling of just wonder looking down on it. And I had that sense of awe. I’ve had that numerous times in my life. It could be with people if you are at the birth of one of your kids. But with me, it’s primarily out when I’m outdoors.

Keltner and Haidt’s (2003) seminal work on awe refers to an awe experience beyond one’s current thinking and comprehension (a sense of vastness), one that therefore requires a new understanding to create (need for accommodation) based on that experience. Awe experiences also involve intense emotions, which often include people describing them as beautiful (Thompson, 2022c). Expanding on the emotional aspect of awe, they were asked to return to the question of how they would explain what awe is, and specifically other, related feelings and emotions associated with an awe experience:

Joy, I guess would be a good one. The freedom from being worried or being concerned about the future or the past. [Awe] inspires you to live in the here and now, to be in the moment, to be centered.

So, I guess that goes along with mindfulness training is helpful. So being mindful. Most of us live, I’m the same way, most of us are either worried about the past even though we cannot change it or [are] concerned about the future when we do not know really what’s going to happen and we might not live to see the future.

And so, I think that feeling, in a good sense of exhilaration, a sense of being unencumbered, sense of freedom, freeing your mind from all the trappings of worries that are on your shoulders even just temporarily, even if it’s just for a few minutes.

It is important to examine the above statement in the context of their specific NASA-related work and the stressors associated with it. Among other tasks, they are in charge of assessing and maintaining the mental health of astronauts. Stressors and high expectations are built into their daily work. Awe experiences and reflecting on past awe moments do not make those stressors disappear.

What awe can do, and as described by the space expert, is provide a much-needed break. Previous awe and resilience research has shown that when perceived as a type of mindfulness practice (Allen, 2018; Tabibnia, 2020; Thompson, 2022a), awe can reduce stress while increasing positive emotions in that moment. This positive impact is not limited to the moment either: it has been shown to persist after the awe moment has passed (Bai et al., 2021; Thompson, 2022c). Importantly, and especially related to their NASA role, as previously stated, awe can enhance a person’s focus, decision-making, critical thinking, creativity, and patience. All these traits are necessary for the participant to excel in their various roles within NASA. This demonstrates the dual benefits of experiencing awe: it can support a person’s personal wellbeing while also enhancing their professional work.

These positive attributes of awe support the argument that awe can be a beneficial practice for people working in high-pressure environments, such as the one the NASA employee works in, yet it can also be applicable to other high-pressure work environments and professions, among them police, other first responders, medical and emergency room personnel, and the military. Previous studies have highlighted awe as a practice to support police personnel (Thompson, 2022a,c, 2023a; Thompson et al., 2022), and our NASA expert confirms this can be the case for other professionals, especially in high-pressure and stressful environments.

Awe has been described as a type of mindfulness practice that is easier to practice than others (D’Ardenne, 2019). The NASA expert explained how awe can serve as a mindfulness moment, even briefly, while also offering a temporary reprieve from life’s stressors. They added that although the awe experience and corresponding emotions are intense, it is still comforting:

I would say that they are very intense … awe fills you up, but fills you up in a way that does not strike your autonomic nervous system in a jarring way. It’s relaxing. That sense of awe that I’m describing is something that you can feel and it’ll help you to relax. That feeling of, I would say, gratitude, I forgot to let that out. [Awe and] gratitude I think go hand in hand.

As they concluded the above statement, they linked awe experiences with another critical resilience practice: gratitude. The relationship between awe, gratitude, and resilience has previously been established (Thompson et al., 2022), while it has also been strongly linked with awe moments (Thompson, 2022c).

In the following passage, the NASA expert responded to being asked what role, both individually and collectively, does awe play in their work:

I think [awe] ties a lot of what we do together because we realize what we are doing has a greater impact that will last longer than … that NASA is going to outlast me in my life. And what I have been a part of is still going to be looked at as being a pioneer in the space program. Multiple space missions that I’ve been personally a part of are the first that have ever been done.

Now later on, let us say 50 years from now, 100 years from now, things are going to be much different, but it’s going to be because of all the work that we are doing now. So, the thought that, yeah, I’m building the foundation of something that’s going to be long lasting and not going to just go away.

I’m grateful that I have a job where I’m not just earning money. I’m doing something that’s way beyond just coming here to get paid, way beyond that.

In this complex passage, they reveal an awareness of the meaning and purpose of their work and the far-reaching impact it has and will have. In addition, they demonstrated a sense of connectedness that often accompanies awe. This sense of connectedness, however, is not limited to those around them today. They clearly see that this connectedness will extend well beyond their own lifetime. Furthermore, the prosocial and self-transcendent attributes of awe are closely linked with this connectedness, as they realize their work contributes to the betterment of mankind, both on this planet and beyond. This unique and rare opportunity is not lost on the NASA expert, who related all this with gratitude.

When asked to reflect on daily moments, which now, while being prompted, could be viewed as moments of awe, they shared the following:

We’re in a lot of meetings. There’s budget planning. Now because I’m in a senior leadership role, there’s a lot of meetings that are draining and I have to pay attention to things that are mundane, right? Because I have a responsibility for a portion of the organization. However, looking at my schedule, on Wednesday I have a meeting with an astronaut…

I think to myself, wow, having that connectedness with somebody that’s a national asset, IQ of 170, has been on the space station on the space shuttle, has a sense of humor, is an all-American person. It’s like, I cannot believe that I get to have that type of an interaction.

Previous research has established that often when asked to describe awe, similar phrases or statements, such as “wow,” are used to denote the profound effect of awe (Thompson, 2022d NARR; Thompson, 2022c). Although this interaction with the astronaut is a required task of theirs, they also demonstrated a genuine curiosity when talking with that individual. Curiosity can often be the result of experiencing awe (Anderson et al., 2020) and is often associated with effectiveness in numerous professions, especially leadership roles (Thompson, 2022a):

I’m like, I cannot wait. I cannot wait to talk to them and to hear how the last year has gone for them. How’s their family? I heard that this individual, for instance, is interested in a space flight and maybe has the opportunity to get a one-year mission in the future, for example.

In addition to curiosity, humility is another attribute of experiencing awe and is related to effective professionals (Stellar et al., 2018; Thompson, 2022a); they displayed a sense of humility once again by acknowledging that they too can take things for granted at times:

There have been times, though, where my mindset has not been right and I either take it for granted or look at that as a burden because it adds more work. My schedule’s pretty busy, but knowing that it’s there, I’m thinking that’s going to be great. I cannot wait.

When prompted if they had ever thought about awe in the context of their work, they reflected on how it is connected to self-awareness:

Well, I do not think I’ve thought about it from the point of dissecting it and looking at it and looking at the word and the definition. But I’m very well aware of my feelings for those times in which I felt that way.

Awe researcher Marianna Graziosi has previously explained there are two types of awe experiences (2018). The first is awe as an expected response to something extraordinary. An example could be a visit to the Great Barrier Reef or witnessing the Northern Lights. Importantly, though, as the NASA expert explains, experiencing awe can also be an extraordinary response to something ordinary:

There are times in which I’ll either drive into work or leave work and it might be just a regular day, right? A regular day. But going into work and seeing the big sign, the [NAME] Space Center or leaving work. And it might be 6:30 at night, almost seven o’clock and my car might be the last one in the parking lot, leaving the building and feeling that sense of, hey, this was a good day. I still love working here.

Graziosi further elaborated this awe concept, as described above by the participant, in terms of the important role of conditioning or having the right frame of mind being just as important as the conditions or the environment. Also, as explained above, when related to one’s work, awe can also support other resilience and wellbeing practices, including finding meaning and purpose in life. The space expert elaborated on this as follows:

I could retire [soon] with a full retirement. But unless something really goes wrong in my life personally, or my [spouse] says, “Hey, I really want to move somewhere else,” or whoever the president is at the time just guts the space program budget, this is the type of job that people just work in until they have really had enough, until they have had their fill.

I could picture easily working here another decade, easy, because it’s not a job that punishes you, it does not drain you, it does not make you feel less than who you are. It actually makes you feel better about yourself. And there’s not a lot of jobs that are like that.

I could picture easily working here another decade.

It is worth noting yet again the sense of gratitude felt by the NASA leader vis-à-vis their work and specifically being part of NASA. A sense of gratitude contributes to a person enjoying work and finding meaning in what they do. Finding meaning and purpose in life is not limited to one’s place of employment; for some, it is found outside their employment.

Additionally, a common component of awe is spirituality. Here, they reflect on both the significance of their faith and, as much as they clearly find meaning in their work, they are also finding their purpose beyond NASA:

I recently have become more attuned to my religious faith and obligations. And rather than hold myself back in my talents that I feel God has given me, I’ve released them. I used to work a lot extra. I actually still work … with underserved populations.

They further explained the significance of this volunteer work to them:

One day I left here and I was having a terrible day as we all do, right? And when I got there [the place they do their volunteer work] and I was listening to what they are going through and knowing that I was there to actually lend myself to them, I suddenly felt great again. And in fact, I’ve had awe moments there. Awe moments of extending myself to people that otherwise would not have anything.

The above passage could be termed a type resilience practice that is described as having both a sense of agency and cognitive reappraisal. Cognitive reappraisal refers to reinterpreting a situation (Southwick and Charney, 2018). Additionally, experiencing awe has been described as a self-transcendent emotion (Yaden et al., 2017; Chirico and Yaden, 2018; Thompson, 2022d), meaning that aside from the personal benefits one gains when experiencing awe, the positive impact is not limited to the individual. Instead, experiencing awe can motivate a person to want to help and support others.

Self-care, regardless of the work or life environment, is critical to being effective at work and to an individual’s personal wellbeing. There is a variety of practices one can engage in to maintain a positive mental and physical healthy life. When asked about how they practice self-care, getting sufficient sleep was mentioned first:

It’s not a job that punishes you, it does not drain you, it does not make you feel less than who you are… If I’m feeling weary a lot of the time, it’s just because I’m older. I’m older than I was 15 years ago when I had more energy. And if I do not sleep well, the next day I do not feel as good as I did, like, 20 years ago. But it’s not because of the job. The job actually makes me feel better.

A sufficient amount of quality sleep is necessary for overall health, while sleep disruptions, specifically of the circadian rhythm, have been linked to numerous mental health issues (Alachkar et al., 2022). Moreover, deficient sleep impairs both short-term and long-term memory, the ability to plan and coordinate activities, and increases the brain’s workload (Dean, 2022). The NASA leader spoke about the importance of getting sufficient sleep:

I also protect my sleep more than I used to. I have a Fitbit next to my phone. Before, I used to not pay that any mind, but now I really pay more attention to making certain that I sleep adequately. And that’s really helped tremendously because when I was young, I did not pay much attention to that. I would wear myself down, but because my body was younger, it did not seem like it needed it.

Lastly, the NASA expert shared the following concluding comment as the interview ended: “I enjoyed our conversation today. I really appreciate it. It made me feel better.”

It has been suggested in previous research (Cuzzolino, 2021; Thompson, 2022d; Thompson and Jensen, 2023), and this study further argues this, that participating in this type of study involving awe and resilience interview can itself be a type of resilience and wellbeing practice. To further examine this idea, an eight-question follow-up survey was sent to the NASA expert, who completed it 19 days after the interview was conducted.

### Post-interview reflections

The post-interview survey consisted of both Likert scale and open-ended questions that allowed the NASA expert to provide a written response. Table 2 displays the Likert scale questions and responses as well as short answers to selected questions. Additional questions and answers are provided later in this section.

**Table 2:** Post-survey answers.

Question	Answer
Would you recommend others in your profession to participate in this study? (Likert scale, 1–5)	*Definitely (5)*
How would you describe participating in the interview?	*Very satisfied (5)*
Please briefly describe what it was like participating in the interview.	*The interview was pleasant and also informative. I enjoyed the experience.*
Overall, if there was anything specific you enjoyed about participating, briefly describe it.	*It was nice to contemplate why working at NASA is so awe inspiring!*

The survey also explored if participating in the study impacted them after the interview was over. Now that time has elapsed since the interview, the NASA scientist was asked to further reflect on awe, “I’ve taken a closer look at what working at NASA means to me and to the world as a whole and have an even greater appreciation for such work.”

Their response demonstrates how participating in the study had a lasting impact beyond simply taking part in the interview. Furthermore, sharing their experiences has a continuing effect, or what has been described as an “upward spiral” of a lasting, positive impact. In this case, the impact was related to their work and finding meaning and purpose in what they do for a living. Additional resilience attributes they displayed included gratitude and a sense of connectedness to the greater world.

Lastly, and again to measure if participating in the study had a lasting influence on them, the last question asked if their perception of awe has changed since the interview concluded, “Yes, I have a broader view and understanding of awe and how it can benefit me and others as well.”

The NASA expert has again shown how, for them, awe can be an epistemic emotion by promoting open-mindedness and filling gaps in knowledge. Additionally, their concluding answer validates how previous studies suggest that awe can be self-transcending by realizing that the benefits of experiencing awe are not limited to them but extend to others as well.

## Discussion

Having a meaning and purpose in life enhances our connectedness to others and allows us to feel as if we are contributing to something beyond ourselves. Reflecting on awe experiences allows a narrative to be constructed that is initially supportive of the individual’s mental health and then extends outward to others. The NASA mental health expert has demonstrated in this study that their awe narrative can contribute to enhancing their sense of meaning and purpose while also benefiting many other resilience skills such as cognitive reappraisal, gratitude, and optimism. These benefits have a sustaining impact that can also extend to others.

This study advances what previous awe research has explored in multiple ways. First, it provided a personal perspective on common awe themes. Second, it further established the relationship between awe and other resilience skills and practices. Third, the data collection method, *via* a semi-structured interview, shows that this type of study can also be utilized as an evidence-based, practical method of enhancing a person’s resilience and mental health.

### Practical implications

In embracing concepts of narrative medicine, this study also aimed to support the participant’s ability to help others in relation to their NASA leadership, medical, and mental health work. The study’s design shows that participating in the study is itself a practice in evoking awe and enhancing resilience while also supporting leadership traits. Thompson and Jensen (2023) demonstrated the value that this type of study can have with hostage negotiators. The present study has advanced their work by showing it can also support professionals in medicine and mental health in examining themselves in order to better serve others. As noted previously, awe has been shown to promote prosocial behaviors, open-mindedness, patience, and other skills needed not only to be an effective mental health and medical professional, but also to benefit one’s own wellbeing.

## Conclusion

This exploratory study provides promising findings that both individuals and medical organizations can embrace with respect to developing and enhancing resilience, providing meaning and purpose (in work and their personal lives), while also contributing to gratitude, social connectedness, and their overall wellbeing. For example, reflective and narrative practices for leadership, as well as the entire workforce, can be incorporated into other trainings while also serving as an independent, brief practice as well.

Additional studies, both quantitative and qualitative, should be carried out to examine the ways awe can be elicited, the impact awe can have on the individual and others, and the extent of the lasting nature of that impact. This study has shown how for one individual, a NASA leader responsible for the wellbeing of astronauts, reflecting on and sharing awe-related narratives can support their resilience and have a positive impact on their life—at work and beyond. Hopefully this study will inspire future research that will further advance these promising results.

## Data availability statement

The de-indetified data supporting the conclusions of this article will be made available by the author, without undue reservation.

## Ethics statement

The studies involving human participants were reviewed and approved by Lipscomb University. The patients/participants provided their written informed consent to participate in this study.

## Author contributions

The author confirms being the sole contributor of this work and has approved it for publication.

## Conflict of interest

The author declares that the research was conducted in the absence of any commercial or financial relationships that could be construed as a potential conflict of interest.

## Publisher’s note

All claims expressed in this article are solely those of the authors and do not necessarily represent those of their affiliated organizations, or those of the publisher, the editors and the reviewers. Any product that may be evaluated in this article, or claim that may be made by its manufacturer, is not guaranteed or endorsed by the publisher.

## References

[ref1] Agnew T. (2022). Reflective practice 2: improving nurses’ mental health and wellbeing. Nursing Times [Online] 118:6.

[ref2] Alachkar A. Lee J. Asthana K. Vakil Monfared R. Chen J. Alhassen S.. (2022). The hidden link between circadian entropy and mental health disorders. Transl. Psychiatry 12:281. doi: 10.1038/s41398-022-02028-3, PMID: 35835742 PMC9283542

[ref3] Allen S. (2018). The science of awe [white paper]. Greater Good Science Center at UC Berkeley. Available at: https://ggsc.berkeley.edu/images/uploads/GGSC-JTF_White_Paper-Awe_FINAL.pdf

[ref4] Anderson C. L. Dixson D. D. Monroy M. Keltner D. (2020). Are aweprone people more curious? The relationship between dispositional awe, curiosity, and academic outcomes. J. Pers. 88, 762–779. doi: 10.1111/jopy.12524, PMID: 31705660

[ref9001] Bai Y. Maruskin L. A. Chen S. Gordon A. M. Stellar J. E. McNeil G. D.. (2017). Awe, the diminished self, and collective engagement: Universals and cultural variations in the small self. J. Pers. Soc. Psychol. 113, 185–209. doi: 10.1037/pspa0000087 28481617

[ref5] Bai Y. Ocampo J. Jin G. Chen S. Benet-Martínez V. Monroy M.. (2021). Awe, daily stress, and elevated life satisfaction. J. Pers. Soc. Psychol. 120, 837–860. doi: 10.1037/pspa0000267, PMID: 33764120

[ref6] Baumeister R. Vohs K. D. Aaker J. L. Garbinsky E. N. (2013). Some key differences between a happy life and a meaningful life. J. Posit. Psychol. 8, 505–516. doi: 10.1080/17439760.2013.830764

[ref7] Bonanno G. A. (2005). Resilience in the face of potential trauma. Curr. Dir. Psychol. Sci. 14, 135–138. doi: 10.1111/j.0963-7214.2005.00347.x

[ref8] Bonner E. T. Friedman H. L. (2011). A conceptual clarification of the experience of awe: an interpretative phenomenological analysis. Humanist. Psychol. 39, 222–235. doi: 10.1080/08873267.2011.593372

[ref9] Charon R. (2001). The patient-physician relationship. Narrative medicine: a model for empathy, reflection, profession, and trust. JAMA 286, 1897–1902. doi: 10.1001/jama.286.15.1897 11597295

[ref10] Chen S. K. Mongrain M. (2020). Awe and the interconnected self. J. Posit. Psychol. 16, 770–778. doi: 10.1080/17439760.2020.1818808

[ref11] Chirico A. Cipresso P. Yaden D. B. Biassoni F. Riva G. Gaggioli A. (2017). Effectiveness of immersive videos in inducing awe: an experimental study. Sci. Rep. 7:1218. doi: 10.1038/s41598-017-01242-0, PMID: 28450730 PMC5430774

[ref12] Chirico A. Yaden D. B. (2018). “Awe: a self-transcendent and sometimes transformative emotion” in The function of emotions. ed. Lench H. (Cham: Springer)

[ref13] Chirico A. Yaden D. B. Riva G. Gaggioli A. (2016). The potential of virtual reality for the investigation of awe. Front. Psychol. 7:1766. doi: 10.3389/fpsyg.2016.01766, PMID: 27881970 PMC5101419

[ref14] Clay R. A. (2022). Are you experiencing compassion fatigue? APA.org. Available at: https://www.apa.org/topics/covid-19/compassion-fatigue

[ref15] Creswell J. W. (2007). Qualitative inquiry and research design: choosing among five approaches (2nd ed.). Thousand Oaks, California: Sage.

[ref16] Cuzzolino M. P. (2021). “The awe is in the process”: the nature and impact of professional scientists’ experiences of awe. Sci. Educ. 105, 681–706. doi: 10.1002/sce.21625

[ref17] Czyżowska N. Gurba E. (2021). Does reflection on everyday events enhance meaning in life and well-being among emerging adults? Self-efficacy as mediator between meaning in life and well-being. Int. J. Environ. Res. Public Health 18:9714. doi: 10.3390/ijerph18189714, PMID: 34574642 PMC8472181

[ref18] D’Ardenne K. (2019). The impact of awe. ASU News. Available at: https://news.asu.edu/20190103-research-takes-your-breath-away-impact-awe

[ref19] Danvers A. F. Shiota M. N. (2017). Going off script: effects of awe on memory for script typical and -irrelevant narrative detail. Emotion 17, 938–952. doi: 10.1037/emo0000277, PMID: 28230393

[ref20] Dawar R. Rodriguez E. Gawri K. Kwon D. Penedo F. J. (2021). Impact of COVID-19 pandemic on well-being and work-related burnout among healthcare workers at an academic center [poster presentation]. ASCO Annual Meeting [online]. doi: 10.1200/JCO.2021.39.15_suppl.11019

[ref21] Dean J. (2022). Sleep deprivation symptoms: 10 psychological effects. Psy Blog. Available at: https://www.spring.org.uk/2021/08/sleep-deprivation-symptoms-effects.php

[ref22] Frechette J. Bitzas V. Aubry M. Kilpatrick K. Lavoie-Tremblay M. (2020). Capturing lived experience: methodological considerations for interpretive phenomenological inquiry. Int J Qual Methods 19, 160940692090725–160940692090712. doi: 10.1177/1609406920907254

[ref23] Frugé E. Drutz J. (2009). Reflective practice & leadership in medicine & medical education. MedEd Portal 5:3182. doi: 10.15766/mep_2374-8265.3182

[ref24] Geertz C. (1973). Thick description: toward an interpretive theory of culture. 1st ed. New York, NY: Basic Books.

[ref25] Goleman D. Boyatzis R. E. McKee A. (2002) Leadership and emotional intelligence Boston, MA: Harvard Business School Press

[ref26] Graziosi M. (2018). In awe of each other: an exploration of awe in close relationships. [Master’s thesis], University of Pennsylvania. Available at: https://repository.upenn.edu/mapp_capstoneabstracts/156/

[ref27] Graziosi M. Yaden D. (2019). Interpersonal awe: exploring the social domain of awe elicitors. J. Posit. Psychol. 16, 263–271. doi: 10.1080/17439760.2019.1689422

[ref9002] Guba E. G. Lincoln Y. S. (1994). “Competing paradigms in qualitative research” in Handbook of qualitative research. eds. Denzin N. K. Lincoln Y. S. (California: Sage. Thousand Oaks), 105–117.

[ref28] Hanson R. (2018). Resilient: 12 tools for transforming everyday experiences into lasting happiness. New York City: Harmony Books.

[ref29] Herbert C. (2022). Belonging at work: the top driver of employee engagement. Qualtrics XM. Available at: https://www.qualtrics.com/blog/belonging-at-work/

[ref30] Holloway I. (1997). Basic concepts for qualitative research. Wiley-Blackwell, Hoboken, NJ.

[ref31] Huang M. Yang F. (2022). Self-transcendence or self-enhancement: people’s perceptions of meaning and happiness in relation to the self. J. Exp. Psychol. Gen. 152, 590–610. doi: 10.1037/xge0001297 36174172

[ref32] Joiner T. (2005). Why people die by suicide. Cambridge, MA: Harvard University Press.

[ref33] Keltner D. Haidt J. (2003). Approaching awe, a moral, spiritual, and aesthetic emotion. Cogn. Emot. 17, 297–314. doi: 10.1080/02699930302297, PMID: 29715721

[ref34] Kim J. Holte P. Martela F. Shanahan C. Li Z. Zhang H.. (2022). Experiential appreciation as a pathway to meaning in life. Nat. Hum. Behav. 6, 677–690. doi: 10.1038/s41562-021-01283-6, PMID: 35145278

[ref35] Korb A. (2015). The upward spiral: using neuroscience to reverse the course of depression, one small change at a time. Oakland, California: New Harbinger Publications.

[ref36] Koshy K. Limb C. Gundogan B. Whitehurst K. Jafree D. J. (2017). Reflective practice in health care and how to reflect effectively. Int. J. Surg. Oncol. 2:e20. doi: 10.1097/IJ9.0000000000000020, PMID: 29177215 PMC5673148

[ref37] Krenzer W. L. Krogh-Jespersen S. Greenslit J. N. Price A. Quinn K. (2018). Assessing the experience of awe: validating the situational awe scale. Available at: https://psyarxiv.com/dsytn/

[ref38] Krisberg K. (2017). Narrative medicine: every patient has a story. AAMC News. Available at: https://www.aamc.org/news-insights/narrative-medicine-every-patient-has-story

[ref39] Launer J. (2002). Narrative-based primary care. A practical guide. Abington, UK: Radcliffe Medical Press.

[ref40] Li J. J. Dou K. Wang Y. J. Nie Y. G. (2019). Why awe promotes prosocial behaviors? The mediating effects of future time perspective and self-transcendence meaning of life. Front. Psychol. 10:1140. doi: 10.3389/fpsyg.2019.01140, PMID: 31191387 PMC6548882

[ref41] Lincoln Y. S. Guba E. G. (1985) Naturalistic inquiry. SAGE, Thousand Oaks, CA, 438–439.

[ref42] Martela F. Ryan R. M. Steger M. F. (2018). Meaningfulness as satisfaction of autonomy, competence, relatedness, and beneficence: comparing the four satisfactions and positive affect as predictors of meaning in life. J. Happiness Stud. 19, 1261–1282. doi: 10.1007/s10902-017-9869-7

[ref43] Meng L. Wang X. (2022). Awe in the workplace promotes prosocial behavior. PsyCh J. 12, 44–53. doi: 10.1002/pchj.593 36058883

[ref44] Mental Health America (n.d.). The mental health of healthcare workers in COVID-19. Available at: https://mhanational.org/mental-health-healthcare-workers-covid-19

[ref45] Mills A. J. Durepos G. Wiebe E. (Eds.). (2010). Thick description. Thousand Oaks, CA: SAGE Publications, Inc.

[ref46] Neubauer B. E. Witkop C. T. Varpio L. (2019). How phenomenology can help us learn from the experiences of others. Perspect. Med. Educ. 8, 90–97. doi: 10.1007/s40037-019-0509-2, PMID: 30953335 PMC6468135

[ref47] NIOSH (2022). Health worker mental health. CDC.gov. Available at: https://www.cdc.gov/niosh/newsroom/feature/health-worker-mental-health.html#:~:text=22%25%20of%20healthcare%20workers%20experienced,%25%20were20women20%5B1%5D.

[ref48] Olazagasti C. Velazquez A. I. Duma N. (2021). Tackling burnout: an endemic problem in the medical field. ASCO Daily News. Available at: https://dailynews.ascopubs.org/do/tackling-burnout-endemic-problem-medical-field

[ref9003] Panchal N. Saunders H. Rudowitz R. Cox C. (2023). The implications of COVID-19 for mental health and substance use. Kaiser Family Foundation. https://www.kff.org/coronavirus-covid-19/issue-brief/the-implications-of-covid-19-for-mental-health-and-substance-use/

[ref49] Piff P. K. Dietze P. Feinberg M. Stancato D. M. Keltner D. (2015). Awe, the small self, and prosocial behavior. J. Pers. Soc. Psychol. 108, 883–899. doi: 10.1037/pspi0000018, PMID: 25984788

[ref50] Rivera G. N. Vess M. Hicks J. A. Routledge C. (2019). Awe and meaning: elucidating complex effects of awe experiences on meaning in life. Eur. J. Soc. Psychol. 50, 392–405. doi: 10.1002/ejsp.2604

[ref51] Roberts C. (2008). Developing future leaders: the role of reflection in the classroom. J. Leadersh. Educ. 7, 116–130. doi: 10.12806/V7/I1/AB1

[ref52] Routledge C. FioRito T. A. (2021). Why meaning in life matters for societal flourishing. Front. Psychol. 11:601899. doi: 10.3389/fpsyg.2020.601899, PMID: 33519608 PMC7842113

[ref53] Rudd M. Vohs K. D. Aaker J. L. (2012). Awe expands people’s perception of time, alters decision making, and enhances well-being. Psychol. Sci. 23, 1130–1136. doi: 10.1177/0956797612438731, PMID: 22886132

[ref54] Shiota M. N. Keltner D. Mossman A. (2007). The nature of awe: elicitors, appraisals, and effects on self-concept. Cognit. Emot. 21, 944–963. doi: 10.1080/02699930600923668

[ref55] Smith J. A. Flowers P. Larkin M. (2009). Interpretative phenomenological analysis: theory, method and research. Thousand Oaks, California: Sage.

[ref56] Smith J. A. Nizza I. E. (2021). Essentials of interpretative phenomenological analysis. Washington, D.C: American Psychological Association.

[ref57] Smith J. A. Osborn M. (2003). “Interpretative phenomenological analysis” in Qualitative psychology: a practical guide to research methods. ed. Smith J. A. (Thousand Oaks, CA: Sage), 51–80.

[ref58] Smith J. A. Osborn M. (2004). “Interpretative phenomenological analysis” in Doing social psychology research. ed. Breakwell G. M. (Oxford, England: British Psychological Society; Blackwell Publishing), 229–254.

[ref59] Southwick S. M. Charney D. S. (2018). Resilience: the science of mastering life’s greatest challenges. 2nd ed. Cambridge, England: Cambridge.

[ref60] Stellar J. E. (2021). Awe helps us remember why it is important to forget the self. Ann. N. Y. Acad. Sci. 1501, 81–84. doi: 10.1111/nyas.14577, PMID: 33547655

[ref61] Stellar J. E. Gordon A. Anderson C. L. Piff P. K. McNeil G. D. Keltner D. (2018). Awe and humility. J. Pers. Soc. Psychol. 114, 258–269. doi: 10.1037/pspi0000109, PMID: 28857578

[ref62] Strumm B. (2022). Reflection for well-being: the reflective practice experiences of social workers employed in global development. Reflective Pract. 24, 238–250. doi: 10.1080/14623943.2022.2158798

[ref63] Sturm V. E. Datta S. Roy A. R. K. Sible I. J. Kosik E. L. Veziris C. R.. (2020). Big smile, small self: awe walks promote prosocial positive emotions in older adults. Emotion 22, 1044–1058. doi: 10.1037/emo0000876, PMID: 32955293 PMC8034841

[ref64] Summers R. F. Gorrindo T. Hwang S. Aggarwal R. Guille C. (2020). Well-being, burnout, and depression among North American psychiatrists: the state of our profession. Am. J. Psychiatry 177, 955–964. doi: 10.1176/appi.ajp.2020.19090901, PMID: 32660300

[ref65] Suzuki W. A. Feliú-Mójer M. I. Hasson U. Yehuda R. Zarate J. M. (2018). Dialogues: the science and power of storytelling. J. Neurosci. Off. J. Soc. Neurosci. 38, 9468–9470. doi: 10.1523/JNEUROSCI.1942-18.2018, PMID: 30381438 PMC6209845

[ref66] Tabibnia G. (2020). An affective neuroscience model of boosting resilience in adults. Neurosci. Biobehav. Rev. 115, 321–350. doi: 10.1016/j.neubiorev.2020.05.005, PMID: 32522489

[ref9004] Thompson J. (2015). Nonverbal communication and the skills of effective mediators: Developing rapport, building trust, and displaying professionalism. Doctoral dissertation, Griffith University.

[ref9005] Thompson J. (2020). Enhancing resilience during the COVID-19 pandemic: A thematic analysis and evaluation of the warr;or21 program. J. Community Safety Wellbeing. 5, 51–56. doi: 10.35502/jcswb.134

[ref67] Thompson J. (2022a). Awe: helping leaders address modern policing problems. J. Community Safety Well-Being 7, 53–58. doi: 10.35502/jcswb.239

[ref68] Thompson J. (2022b). Your daily dose of awe: accessing real resilience. Psychology Today. Available at: https://www.psychologytoday.com/us/blog/beyond-words/202201/your-daily-dose-awe-accessing-real-resilience

[ref69] Thompson J. (2022c). Enhancing resilience: an interpretative phenomenological analysis of the awe project. J. Community Safety Well-Being 7, 93–110. doi: 10.35502/jcswb.265

[ref70] Thompson J. (2022d). Awe narratives: a mindfulness practice to enhance resilience and wellbeing. Front. Psychol. 13:840944. doi: 10.3389/fpsyg.2022.840944, PMID: 35496194 PMC9049271

[ref71] Thompson J. (2022e). Investigating police suicide: how the psychological autopsy can provide clarity. Police Chief Magazine, 14–16. Available at: https://www.policechiefmagazine.org/nimble-auth/login/?redirect_to=https%3A%2F%2Fwww.policechiefmagazine.org%2Ffocus-on-officer-wellness-investigating-police-suicide%2F

[ref72] Thompson J. (2023a). Homicide & special victim investigators: experiencing awe to support investigations & personal wellness. IACP Police Chief Magazine. Available at: https://www.policechiefmagazine.org/experiencing-awe/

[ref73] Thompson J. (2023b). Narrative health: examining the relationship between the phenomenon of awe with resilience and wellbeing. J. Community Safety Wellbeing.

[ref74] Thompson J. Drew J. M. (2020). Warr;or21: a 21-day program to enhance first responder resilience and mental health. Front. Psychol. 11. doi: 10.3389/fpsyg.2020.02078, PMID: 33013529 PMC7505768

[ref75] Thompson J. Grubb A. R. Ebner N. Chirico A. Pizzolante M. (2022). Increasing crisis hostage negotiator effectiveness: embracing awe and other resilience practices. Cardozo J. Conflict Resol. 23, 615–685. Available at: https://static1.squarespace.com/static/60a5863870f56068b0f097cd/t/62c5bb989716c4185529716a/1657125784611/CAC309_crop.pdf

[ref76] Thompson J. Jensen E. (2023). Hostage negotiator resilience: a phenomenological study of awe. Front. Psychol. 14:1122447. doi: 10.3389/fpsyg.2023.1122447 37113118 PMC10127252

[ref77] Tongeren D. R. Green J. D. Davis D. E. Hook J. N. Hulsey T. L. (2016). Prosociality enhances meaning in life. J. Posit. Psychol. 11, 225–236. doi: 10.1080/17439760.2015.1048814

[ref78] van Manen M. (1990). Researching lived experience: human science for an action sensitive pedagogy. New York: State University of New York Press.

[ref79] Van Orden K. A. Cukrowicz K. C. Witte T. K. Joiner T. E. (2012). Thwarted belongingness and perceived burdensomeness: construct validity and psychometric properties of the interpersonal needs questionnaire. Psychol. Assess. 24, 197–215. doi: 10.1037/a0025358, PMID: 21928908 PMC3377972

[ref80] Van Orden K. A. Witte T. K. Cukrowicz K. C. Braithwaite S. R. Selby E. A. Joiner T. E. Jr. (2010). The interpersonal theory of suicide. Psychol. Rev. 117, 575–600. doi: 10.1037/a0018697, PMID: 20438238 PMC3130348

[ref81] Walker J. Gilovich T. (2021). The streaking star effect: why people want superior performance by individuals to continue more than identical performance by groups. J. Pers. Soc. Psychol. 120, 559–575. doi: 10.1037/pspa0000256, PMID: 32790471

[ref82] Williams L. (2019). Having a sense of meaning in life is good for you. So how do you get one? Available at: https://newsroom.unsw.edu.au/news/health/having-sense-meaning-life-good-you-so-how-do-you-get-one

[ref83] Yaden D. B. Haidt J. Hood R. W. Vago D. R. Newberg A. B. (2017). The varieties of self-transcendent experience. Rev. Gen. Psychol. 21, 143–160. doi: 10.1037/gpr0000102

[ref9006] Yaden D. B. Kaufman S. B. Hyde E. Chirico A. Gaggioli A. Zhang J. W.. (2019). The development of the Awe Experience Scale (AWE-S): A multifactorial measure for a complex emotion. J. Posit. Psychol. 14, 474–488. doi: 10.1080/17439760.2018.1484940

[ref84] Zaharias G. (2018). What is narrative-based medicine? Narrative-based medicine 1. Can. Fam. Physician 64, 176–180. PMID: 29540381 PMC5851389

